# Secondary Syphilis with Pleural Effusion: Case Report and Literature Review

**DOI:** 10.1155/2012/409896

**Published:** 2012-12-13

**Authors:** Abdel-Naser Elzouki, Mustafa Al-Kawaaz, Zaid Tafesh

**Affiliations:** ^1^Department of Medicine, Weill Cornell Medical College of Qatar, Hamad General Hospital, Hamad Medical Corporation, P.O. Box 3050, Doha, Qatar; ^2^Weill Cornell Medical College of Qatar, Hamad General Hospital, Hamad Medical Corporation, Doha, Qatar

## Abstract

Here we present a case of a 38-year-old Indian man with a history of extramarital relationships who presented with pleurisy, skin rash, and radiological findings of pleural effusion. After thorough investigation of the etiology of his acute illness, he was found to be positive for syphilis. Review of literature revealed a small number of case reports of pleural effusion as a manifestation of secondary syphilis. The review of criteria proposed in the literature was utilized to diagnose this patient as a case of pulmonary syphilis.

## 1. Introduction

Syphilis is a sexually transmitted illness caused by the organism *Treponema pallidum*. The infection itself is acquired mainly via sexual contact with clinical manifestations such as a chancre or skin rash. However, although less common, the infection can also be contracted by nonsexual contact, organ transplantation, blood transfusion, or in utero infection [1]. With the introduction of penicillin therapy to counter syphilis infection, the number of cases in the developed countries such as United States has decreased by 95% since 1943 [1].

The natural course of syphilis begins with an inoculation period of 21 days on average, rarely exceeding 6 weeks. Syphilis can present itself in three different stages, specifically primary, secondary, and tertiary syphilis, with each stage characterized by unique clinical manifestations.

Although uncommon, report of pulmonary illness in patients with proven syphilis exists in the literature. Only a handful of cases report such involvement, with variable presentations reported. Pulmonary syphilis occurred mainly in congenital and tertiary syphilis in the preantibiotic era, but, since 1967, it has been occurring mainly during secondary syphilis [2]. David and his coworkers [2] sited Coleman et al. [3] regarding the fact that lung involvement in patients with syphilis during the preantibiotic area ranged from 1% to 12.5%.

Here we present a case of a patient with suspected pulmonary syphilis, with significant pleural effusion and clinical and diagnostic evidence of syphilis infection. Our case is consistent with the fact that most infections are now related to cases resembling secondary syphilis. We chose to incorporate the diagnostic criteria for pulmonary syphilis proposed by Coleman et al which was presented by David et al. [2].

## 2. Case Report

A 38 year-old Indian man presented with a past medical history significant for recurrent low-grade fevers and a new diagnosis of Diabetes Mellitus being treated with metformin. He complained of pain in the right chest and right upper abdomen for 10 days associated with dyspnea. His pain was sharp in nature and tearing in quality. He described it as a localized type of pain that was nonradiating, which began intermittently and progressed to severe constant pain until his presentation. He graded his pain as 7/10 in severity. The pain was aggravated by movement and deep inspiration and was alleviated by over-the-counter analgesics. He reports no history of similar episodes/symptoms prior to the 10 days before admission. Five days prior to admission his pain worsened and he sought medical attention at a primary health care clinic in Doha, Qatar, where he was given intramuscular analgesics, which reduced the pain over the next 2 days. Afterwards, he began to suffer from the pain described above and presented to the Hamad General Hospital Accidents and Emergency (A/E) Department. The patient remained in the A/E department for one day awaiting an available bed after which he was admitted to the medical ward with the preliminary diagnosis of community-acquired pneumonia. The patient had no history of cough or hemoptysis, no history diarrhea or constipation, no hematochezia, no melena, no hematemesis or dysphagia or vomitting. He had no hematuria, no dysuria or any change in urine color or frequency. He had no history of contact with sick people, no history of recent travel, and no history suggestive of asbestos exposure. On questioning, the patient reported a history of extramarital sexual relationships in the past.

**Figure 1:**
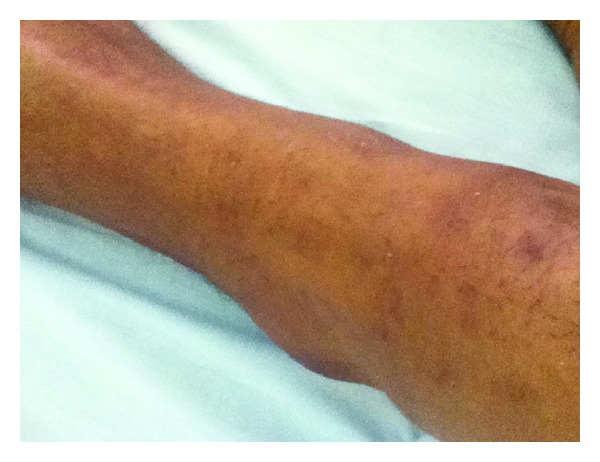
Patchy hyperpigmentations on the patient's shin.

On physical examination the patient's vitals on admission were a temperature of 37.2 degrees Celsius, a heart rate of 110 beats per minute, a blood pressure of 130/80 mmHg, and a respiratory rate of 40 breaths per minute. General and systemic examinations were significant for a patchy, macular hyperpigmented rash on the dorsum and pedal areas of his foot and on his shins bilaterally (Figure 1) and both decreased breath sounds and vocal fremitus in the right lower chest region.

**Figure 2:**
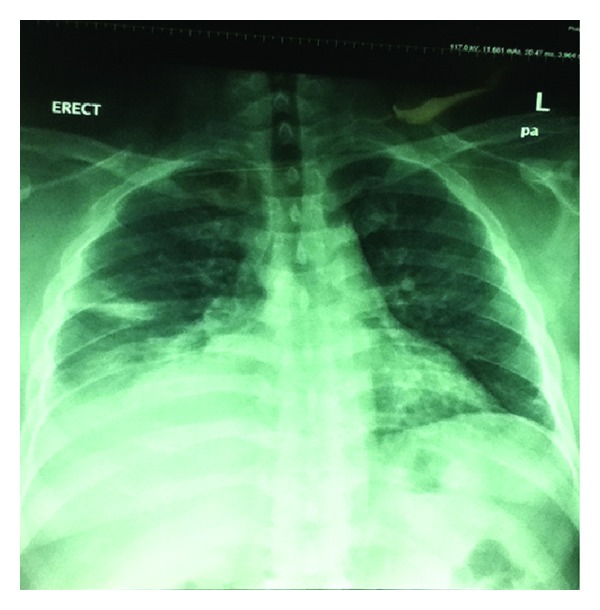
Chest X-ray on admission showed infiltrates and pleural effusion on the right lower zone.

**Figure 3:**
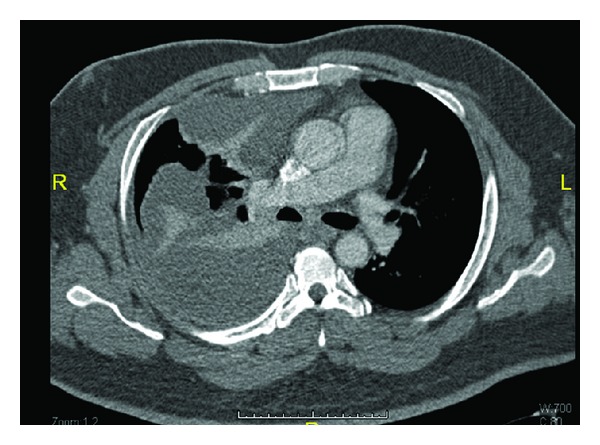
Chest CT showing the encysted pleural effusion.

A complete blood cell count revealed a white blood cell count of 12.3 × 10^3^/*μ*L, Hemoglobin of 15 g/dL, and platelet count of 219 × 10^3^/*μ*L. Kidney function tests were notable for a blood urea nitrogen of 4.3 mmol/L and a creatinine level of 69 *μ*mol/L. Chest X-ray revealed patchy infiltrates on the right lower zone and obliteration of the right costophrenic angle suggesting pleural effusion (Figure 2). Pleural fluid aspiration was pale yellow with a white blood cell count of 1260/*μ*L with a differential of 87% neutrophils and 13% lymphocytes, a red blood cell count of 1590/*μ*L, and a pH of 7.237. A rare giant cell was noted on microscopic examination of the fluid. Pleural fluid culture was negative for growth. Pleural biopsy examination was consistent with empyema and negative for granulomas. Quantiferon test on pleural biopsy was negative. CT scan of chest of chest showed multiple areas of encysted pleural effusion on the right lung (Figure 3). Transesophageal echocardiography showed no thrombi or intracardiac masses and ruled out endocarditis.

Serologic testing was also performed and was positive for *Treponema pallidum* antibody, and RPR was reactive 1 : 1. Inno-lia syphilis confirmatory test was also positive. HIV testing was yielded a negative result. Blood, urine, and sputum cultures were all negative. Sputum for acid fast bacilli was also negative. Respiratory panel which includes respiratory syncytial virus PCR, Influenza-A PCR, Influenza-B PCR, Parainfluenza-1 PCR, Parainfluenza 2 PCR, Parainfluenza-3 PCR, Parainfluenza-4 PCR, Coronavirus-NL63 PCR, Coronavirus-COC43, Coronavirus-229E PCR, Rhinovirus PCR, Entero virus PCR, Coronavirus HKU, Human Metapneumovirus PCR, Boca Virus, Parechovirus, Adenovirus PCR, Pandemic Influenza-A H1N1, and *Mycoplasma pneumoniae* is all negative.

**Figure 4:**
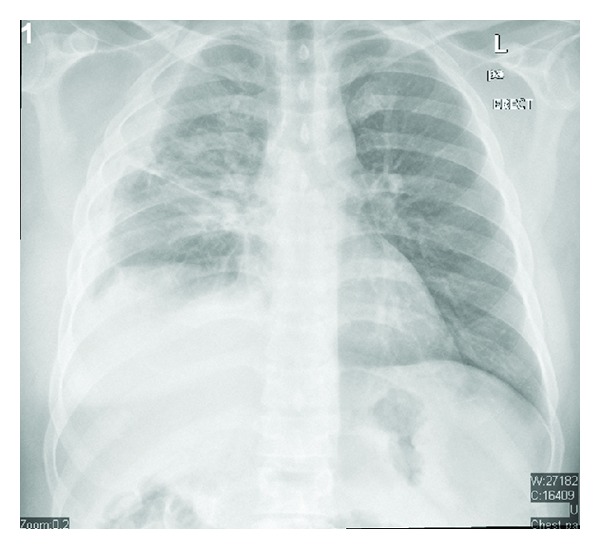
Chest X-ray on discharge.

The patient was initially diagnosed with community acquired pneumonia complicated by parapneumonic effusion and was given Piperacillin/tazobactam for a week. The patient had repeated fever spikes and thus was switched to meropenem to assess response to another antibiotic. He responded well to Meropenem, and his fever spikes resided and began to show clinical improvement. Regarding his surgical course, a chest tube was inserted to drain the effusion, then it was determined that thoracotomy is essential for total removal of the effusion. The operation was complicated by a hematoma. This hematoma was removed later by a second thoracotomy. The patient thereafter stabilized from a surgical point of view and was discharged in a good condition. His chest X-ray on discharge showed significant improvement (Figure 4).

## 3. Discussion

In a patient with pleural effusion presenting only with pleurisy, skin rash, and positive serology for syphilis, although rare, it is important to consider pulmonary syphilis as one of the differentials. In our patient syphilis was considered after ruling out most of the common causes of plural effusion. This diagnosis becomes more probable if the patient does not initially respond to wide spectrum antibiotics. Although the patient was receiving Tazocin IV for 10 days, he continuously had fever spikes and complained of fatigue and generalized weakness. Tazocin does not cover syphilis; in fact it has been shown to mask the symptoms of primary and secondary syphilis when high doses are used [4]. The patient improved later after switching him to meropenem, whose coverage includes *Treponema pallidum*.

Based on our review of the English literature, we were able to locate 11 cases of pulmonary involvement in the presentation of secondary syphilis since 1966 [2, 3, 5–13]. Most of these patients had skin rash as one of the presenting symptoms. One of them was negative for HIV, three were positive, and the rest had an unknown status. These patients had variable chest X-ray finding, some associated with pleural effusion. Of these 11 patients those who underwent biopsy (two cases) showed nonspecific granulomas [2, 5]. Our patient had no granulomas on pleural biopsy points to an indirect effect of the spirochetes in creating the pleural effusion.

The positive RPR Inno-lia syphilis confirmatory test ascertains that our patient has syphilis; however, it is difficult to confirm that the lung infiltrates, and pleural effusion is actually due to syphilis. To solve this problem we followed the clinical criteria proposed by Coleman et al. [3] to diagnose pulmonary involvement secondary to syphilis: (1) historical and physical findings typical of secondary syphilis; (2) serologic test results positive for syphilis; (3) pulmonary abnormalities seen radiographically with or without associated symptoms or signs; (4) exclusion of other forms of pulmonary disease, when possible, according to findings of serological tests, sputum smears and cultures, and cytological examination of sputum; (5) response to antisyphilis therapy of signs found by radiological examination [2, 3]. Our patient fulfills all components of this diagnostic criteria for diagnosis for pulmonary syphilis.

In conclusion, a patient presenting with lung infiltrates and pleural effusion that later is associated with fever has a wide differential diagnosis. Often, common causes such as pulmonary tuberculosis, bacterial infection, or neoplasm are investigated. Once investigation fails to describe the etiology of such a patient's presentation, physicians can be left wondering of the underlying cause. However, rare causes of pulmonary infection exist, and, as demonstrated by our patient, syphilis can be included as one such possible cause. As more cases are reported in the future, pulmonary syphilis may find itself higher on the differential diagnosis for such patients, particularly in patients from certain geographical areas where treatment for primary syphilis is not as readily available.
